# Hypothyroidism affects cholelithiasis causally: A two-sample bidirectional Mendelian randomization study

**DOI:** 10.7555/JBR.38.20240264

**Published:** 2025-02-08

**Authors:** Xu Han, Hong Zhu

**Affiliations:** Department of Gastroenterology, the First Affiliated Hospital of Nanjing Medical University, Nanjing, Jiangsu 210029, China

Dear Editor,

Observational studies in epidemiology have identified a correlation between hypothyroidism and cholelithiasis^[1–2]^. However, the causal relationship between the two diseases remains unclear. To investigate the potential causal relationship, we employed a two-sample bidirectional Mendelian randomization (MR) analysis.

The data for hypothyroidism were obtained from the IEU Open GWAS Project (https://gwas.mrcieu.ac.uk; ukb-a-77, *n* = 337159), and the data for cholelithiasis were sourced from the FinnGen biobank (https://r8.finngen.fi/; finngen_R8_K11_CHOLELITH, *n* = 334277).

The detailed procedures of this study are shown in ***Supplementary Fig. 1*** (available online). We used multiple analytical methods, including inverse variance weighted (IVW), MR-Egger regression, weighted median, simple mode, and weighted mode, alongside various sensitivity analyses. To reinforce our findings, we exchanged the exposure and outcome data and subsequently determined the causal effect of cholelithiasis on hypothyroidism following the same procedure.

Initially, a total of 83 single nucleotide polymorphisms (SNPs) were screened from the exposure genome-wide association study (GWAS) dataset (*P* < 5e−8, *r*^2^ > 0.001, 10000 kb). To avoid the weak instrument bias, only 69 SNPs (*F*-statistic > 10) were included in the analysis. We also extracted relevant information for these 69 SNPs from the outcome GWAS dataset, and 66 SNPs were identified. For SNPs missing in the outcome dataset, we used the online tool LDLink (https://ldlink.nih.gov/) to find proxy SNPs (linkage disequilibrium [LD] *r*^2^ > 0.8, present in the outcome dataset, and with consistent alleles). SNPs without suitable proxies were excluded^[3]^. A secondary selection of SNPs was performed after data harmonization. We removed SNPs that had palindromic structures and those marked as "MR-Keep = FALSE" (rs2823272, rs2921053, rs66749983, rs7582694, rs761357, and rs7768019). Through PhenoScanner V2 screening, we excluded SNPs associated with cholelithiasis or known risk factors, including primary sclerosing cholangitis (rs4276275 and rs7090530), body mass index (rs705702), diabetes-related traits (rs12980063, rs229540, rs3184504, and rs3850765), chronic liver disease, and rheumatoid arthritis (rs11052877). We found that all SNPs primarily affected the exposure rather than the outcome based on the Steiger directionality test. Finally, 52 independent and effective SNPs were selected for analysis (***Supplementary Table 1***, available online).

After rigorous MR analyses, we found that hypothyroidism has a strong causal effect on cholelithiasis: MR-Egger (odds ratio [OR] = 4.067, 95% confidence interval [CI]: 1.175–14.082, *P* = 0.031), weighted median (OR = 3.841, 95% CI: 1.667–8.852, *P* = 0.002), IVW (OR = 2.950, 95% CI: 1.663–5.233, *P* < 0.001), and weighted mode (OR = 2.839, 95% CI: 1.136–7.096, *P* = 0.030). However, no significant result was found using the simple mode (*P* = 0.342). IVW is the most important analysis method in MR studies, because it provides an unbiased estimate of causality in an ideal state^[4]^. Although MR-Egger may infer the corrected causality, its statistical efficiency is lower than that of IVW^[5]^. When more than 50% of SNPs are effective, the weighted median results are accurate^[6]^. Although the simple mode provides robustness for pleiotropy, it is not as powerful as IVW^[7]^. The weighted mode is sensitive to the difficult bandwidth selection for mode estimation. When the maximal subset of tools with similar causal effects is valid, the weighted mode results are reliable^[8]^. According to the IVW result, individuals with hypothyroidism-related traits had a 2.95-fold increased risk of developing cholelithiasis, compared with those without hypothyroidism.

We then performed multiple sensitivity analyses to verify the robustness of our results. The results of the heterogeneity test were not statistically significant (*P* > 0.05). The multiplicative random effects model showed a significant association between hypothyroidism and cholelithiasis (*P* < 0.05, *b* > 0). The MR-Egger intercept test (*P* = 0.57) indicated no significant pleiotropic effect of the SNPs. Additionally, we performed the MR-PRESSO test and found no significant outliers.

The scatter plot showed that an increased risk of cholelithiasis was associated with hypothyroidism (***Fig. 1A***). The red line at the bottom of the forest plot indicates that hypothyroidism increases the risk of cholelithiasis in the IVW analysis (***Fig. 1C***). The leave-one-out test showed that all the lines were on the right side of 0, demonstrating the robustness of our results (***Fig. 1D***). The funnel plot was symmetrical, consistent with the results of the heterogeneity test (***Fig. 1E***).

**Figure 1:**
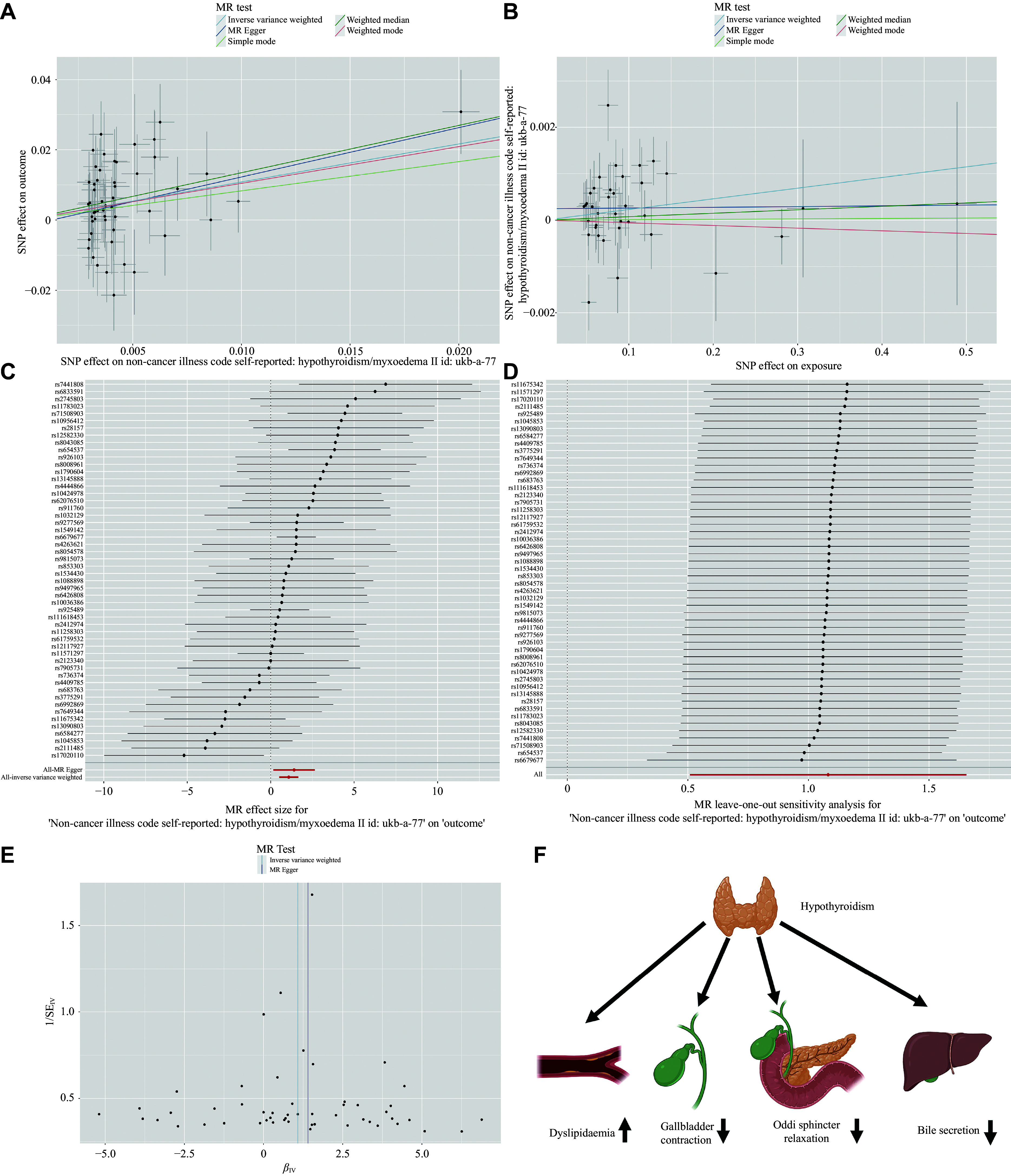
Hypothyroidism affects cholelithiasis causally. A: Scatter plot illustrating the distribution of individual risk ratio estimates for hypothyroidism with cholelithiasis as the outcome. B: Scatter plot illustrating the distribution of individual risk ratio estimates for cholelithiasis with hypothyroidism as the outcome. C: The forest plot shows the effect size of each SNP representing hypothyroidism on cholelithiasis. Each horizontal solid line reflects the result estimated by a single SNP using the Wald ratio method. The lowest red line indicates that hypothyroidism increases the risk of cholelithiasis under IVW. D: Leave-one-out sensitivity analysis of the causal effect of hypothyroidism on cholelithiasis. The red line indicates reliable estimations from the IVW and MR-Egger methods. E: The funnel plot shows the heterogeneity of the Mendelian randomization causal effect of hypothyroidism on cholelithiasis. The points on both sides are roughly symmetrically distributed. F: Four mechanisms by which hypothyroidism increases the risk of cholelithiasis. Abbreviations: MR, Mendelian randomization; SNP, single nucleotide polymorphism; SE, standard error; IV, instrumental variable.

We exchanged the exposure and outcome data to determine the causal effect of cholelithiasis on the risk of hypothyroidism. Following the same steps, a total of 56 SNPs were screened, and only 47 SNPs (*F*-statistic > 10) were included in the current study. After data harmonization and removal of nine SNPs marked as "MR-Keep = FALSE", 38 SNPs were selected. Through PhenoScanner V2 screening, rs174592 (hypothyroidism-related) was removed. Finally, 37 SNPs were selected (***Supplementary Table 2***, available online). The results of MR-Egger (OR = 1.000, 95% CI: 0.996–1.005, *P* = 0.948), weighted median (OR = 1.001, 95% CI: 0.998–1.004, *P* = 0.623), IVW (OR = 1.002, 95% CI: 0.999–1.005, *P* = 0.054), simple mode (OR = 1.000, 95% CI: 0.994–1.007, *P* = 0.978), and weighted mode (OR = 0.999, 95% CI: 0.996–1.003, *P* = 0.767) were analyzed. Because the IVW result was not significant, and the directions of the other methods were inconsistent, the MR analyses of cholelithiasis affecting hypothyroidism were not significant (***Fig. 1B***).

The association between hypothyroidism and cholelithiasis has been controversial for decades. Although numerous observational studies have established an association between the two diseases, which is consistent with our findings^[9–10]^, hypothyroidism increasing the risk of cholelithiasis may be related to several mechanisms, including disorders of lipid metabolism, decreased gallbladder motility, impaired relaxation of the human sphincter of Oddi, and reduced bile secretion (***Fig. 1F***).

First, increasing evidence has demonstrated a strong connection between hypothyroidism and lipid metabolic disorders. Hypothyroidism significantly increases the expression of the *ABCG8* gene in the liver^[10]^, promoting the entry of more cholesterol into bile and directly contributing to cholesterol gallstone formation^[11]^. Moreover, low levels of thyroid hormones decrease the expression levels of hepatic low-density lipoprotein receptors, limiting cholesterol uptake by hepatocytes and resulting in hypercholesterolemia^[12]^. Cholesterol 7α-hydroxylase (CYP7A1) is the rate-limiting enzyme in the liver, which converts cholesterol into primary bile acids. Studies have shown that thyroid hormones may increase *CYP7A1* mRNA expression levels, which is crucial because bile acid synthesis is an important mechanism for the liver to regulate excess cholesterol^[13]^. Interestingly, hypothyroid patients often exhibit elevated thyroid-stimulating hormone levels, which have dual effects on cholesterol metabolism. Elevated thyroid-stimulating hormone levels not only increase serum cholesterol by upregulating HMG-CoA reductase (the rate-limiting enzyme in cholesterol synthesis), but also inhibit the conversion of cholesterol to bile acids through the SREBP-2/HNF-4a/CYP7A1 pathway^[14]^. In addition, the effect of hypothyroidism on renal circulation is noteworthy, as it reduces renal plasma flow and lowers the glomerular filtration rate, which may indirectly influence lipid metabolism^[12]^.

Second, hypothyroidism affects gallbladder motility, although the specific molecular mechanisms remain unclear. The reduced metabolic capacity in hypothyroid patients may impair gallbladder contraction. Additionally, hypercholesterolemia caused by hypothyroidism increases cholesterol levels within gallbladder smooth muscle cells, thereby affecting smooth muscle contraction^[15]^. These factors together compromise efficient gallbladder emptying, potentially exacerbating biliary complications.

Third, hypothyroid patients exhibit a decreased ability of the sphincter of Oddi to regulate bile excretion, increasing the risk of common bile duct stones. This occurs because the sphincter of Oddi's relaxation is partially dependent on thyroid hormone action. In cells of the sphincter of Oddi, thyroid hormones cause cell membrane hyperpolarization by opening adenosine triphosphate-sensitive K^+^ channels, reducing calcium ion influx, and limiting smooth muscle contraction^[16]^. Therefore, the impaired sphincter of Oddi relaxation further disrupts bile flow.

Lastly, hypothyroidism reduces bile secretion by hepatocytes, diminishing the ability to clear biliary sediment^[2]^. Since bile acids are a critical component of bile, we hypothesize that disturbances in bile acid metabolism play a significant role in the reduced bile secretion observed in hypothyroidism. Beyond the previously noted decrease in bile acid synthesis, impairment in bile acid reutilization also warrants attention. Notably, bile acid transporter expression is significantly reduced in the livers of animals with hypothyroidism, disrupting the enterohepatic circulation of bile acids^[12]^. Furthermore, a recent study has demonstrated that hypothyroidism increases the incidence of cholesterol stones in mice by enhancing the hydrophobicity of primary bile acids^[17]^. Despite these findings, the underlying biological mechanisms require further elucidation.

The main advantage of the current study is that we used two-sample bidirectional MR analyses. Because SNPs are theoretically randomly distributed, this significantly reduces the effects of confounding factors and reverse causality^[18]^. Additionally, the data used in the current study were drawn from multiple studies, and the sample size was significantly larger than in any previous observational study, which should increase the accuracy of the results.

However, the current study also has some limitations. First, the individuals we studied were exclusively of European descent, and the findings may not apply to the general population. Second, because we employed several analytical methodologies, there may be some potential multiple-testing effects. Nonetheless, the results of sensitivity analyses demonstrated the reliability of our findings. Third, the current study may only provide a preliminary assessment of the causal relationship between hypothyroidism and cholelithiasis, and further prospective studies are necessary to validate our conclusions.

Collectively, the current study found a strong causal effect of hypothyroidism on cholelithiasis but did not find a similar effect in the opposite direction. These findings may help remind clinicians to pay closer attention to the link between the two diseases.

The current study was sponsored by grants from the Jiangsu Province 333 High-level Talent Training Project (Grant No. LGY2016010), the Nanjing Science and Technology Development Plan (Grant No. 201715003), and the Jiangsu Province Six Talent Peaks (Grant No. WSN-030).

We thank the FinnGen Biobank and the UK Biobank for providing the GWAS data.

Yours sincerely,Xu Han, Hong Zhu^✉^ Department of Gastroenterology, the First Affiliated Hospital of Nanjing Medical University,Nanjing, Jiangsu 210029, China.^✉^Corresponding author: Hong Zhu. E-mail: zhuhong1059@126.com.

## SUPPLEMENTARY DATA

Supplementary data to this article can be found online.
