# Supplementary material for: Hypothyroidism affects cholelithiasis causally: A two-sample bidirectional Mendelian randomization study
Source: J Biomed Res. 2025 Feb 8;39(3):319–24. doi: 10.7555/JBR.38.20240264 (PMC12239981; doi:10.7555/JBR.38.20240264)
Supplement: Supplementary file 1 — Supplementary data to this article can be found online. [file jbr-39-3-319-Supplementary.pdf]

Available online at www.jbr-pub.org.cn

Open Access at PubMed Central

Journal of Biomedical Research, 2025 39(3): 1–2

Supplementary Data

Hypothyroidism affects cholelithiasis causally: A two-sample bidirectional Mendelian randomization study

Xu Han, Hong Zhu✉

Department of Gastroenterology, the First Affiliated Hospital of Nanjing Medical University, Nanjing, Jiangsu 210029, China.

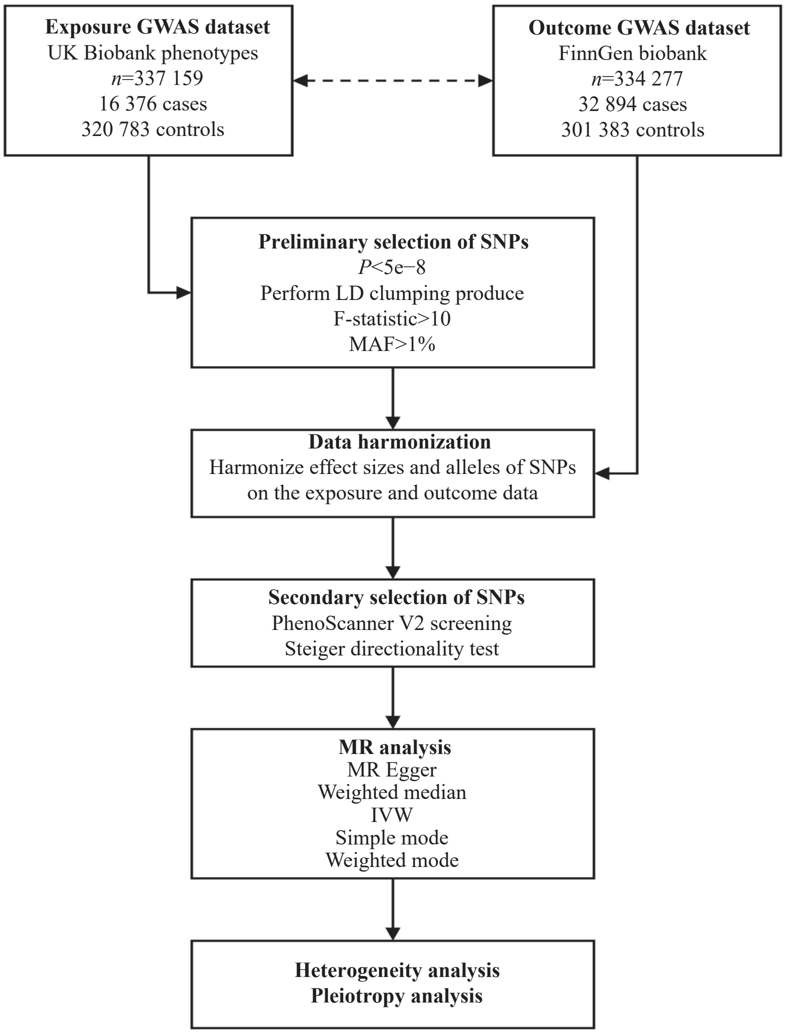

Supplementary Fig. 1 **Overall design of the MR analysis framework in the study.** A flow chart depicts how the MR analysis was conducted step by step in the study. Abbreviation: MR, Mendelian randomization.

✉Corresponding author: Hong Zhu, Department of Gastroenterology, the First Affiliated Hospital of Nanjing Medical University, Nanjing, Jiangsu 210029, China. E-mail: zhuhong1059@126.com.

Received: 19 August 2024; Revised: 02 January 2025; Accepted: 07 January 2025; Published online: 08 February 2025

CLC number: R575.62, Document code: B

The authors reported no conflict of interests.

This is an open access article under the Creative Commons Attribution (CC BY 4.0) license, which permits others to distribute, remix, adapt and build upon this work, for commercial use, provided the original work is properly cited.

© 2025 by Journal of Biomedical Research.

https://doi.org/10.7555/JBR.38.20240264

Supplementary Table 1 (available online) shows SNPs used for analyzing the causal effect of hypothyroidism on cholelithiasis.

Supplementary Table 2 (available online) shows SNPs used for analyzing the causal effect of cholelithiasis on hypothyroidism.
